# Supplementary material for: Empowering the on-site detection of nucleic acids by integrating CRISPR and digital signal processing
Source: Nat Commun. 2024 Jul 25;15:6271. doi: 10.1038/s41467-024-50588-3 (PMC11272939; doi:10.1038/s41467-024-50588-3)
Supplement: Supplementary file 1 — Supplementary Information [file 41467_2024_50588_MOESM1_ESM.pdf]

Empowering the on-site detection of nucleic acids by integrating CRISPR and digital signal processing

Chang Yeol Lee[1,2,3#], Hyunho Kim[1,2#], Ismail Degani[1,4#], Hanna Lee[1], Angel Sandoval[1], Yoonho Nam[1,5], Madeleine Pascavis[1,6], Hyun Gyu Park[5], Thomas Randall[7], Amy Ly[8], Cesar M. Castro[1,9*], Hakho Lee[1,2*]

[1] Center for Systems Biology, Massachusetts General Hospital Research Institute, Boston, MA, USA
[2] Department of Radiology, Massachusetts General Hospital, Harvard Medical School, Boston, MA, USA
[3] Bionanotechnology Research Center, Korea Research Institute of Bioscience and Biotechnology (KRIBB), Daejeon, Republic of Korea
[4] Department of Electrical Engineering and Computer Science, Massachusetts Institute of Technology, Cambridge, MA, USA
[5] Department of Chemical and Biomolecular Engineering (BK21 Four), Korea Advanced Institute of Science and Technology (KAIST), 291 Daehak-ro, Yuseong-gu, Daejeon 34141, Republic of Korea.
[6] CaNCURE program, College of Science, Northeastern University, Boston, MA, USA
[7] Department of Obstetrics and Gynecology, Massachusetts General Hospital, Boston, MA, USA
[8] Department of Pathology, Massachusetts General Hospital, Harvard Medical School, Boston, MA, USA
[9] Department of Medicine, Massachusetts General Hospital, Harvard Medical School, Boston, MA, USA

[#]These authors contributed equally.

Corresponding Authors:
Cesar M. Castro, MD
Hakho Lee, PhD
Center for Systems Biology
Massachusetts General Hospital
185 Cambridge St, CPZN 5206
Boston, MA, 02114
617-726-8226
Castro.Cesar@mgh.harvard.edu
hlee@mgh.harvard.edu

SUPPLEMENTARY FIGURES AND TABLES

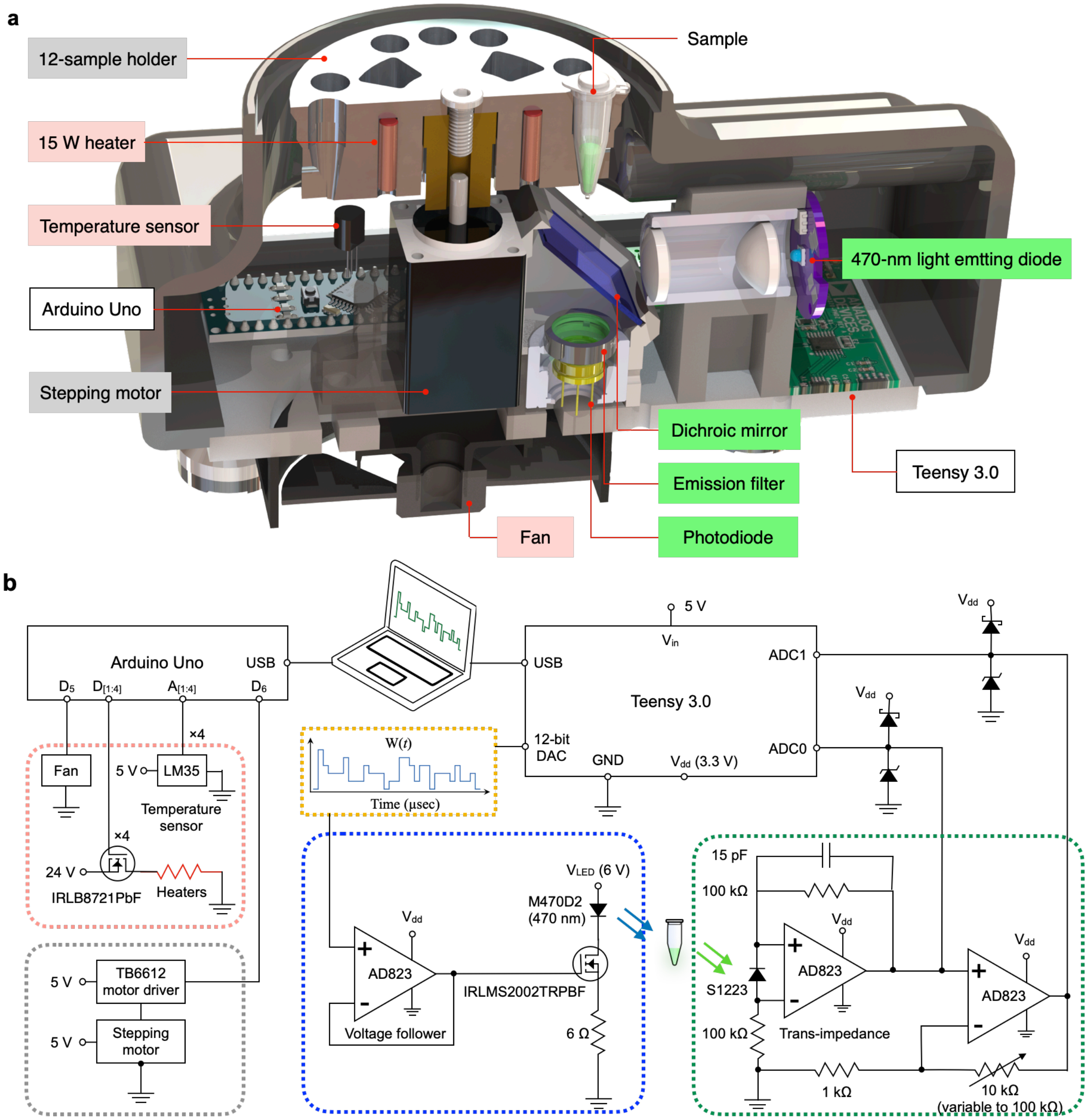

Supplementary Figure 1. Detailed design of the CreDiT device. (a) The mechanical and electronic parts. The device had a module for multi-sample processing (gray boxes), a thermal control unit (pink), fluorescent optics (green), and control boards (white). All parts were integrated into a compact, portable device. **(b)** Electrical circuit diagram of the CreDiT device. Two microcontrollers (Arduino Uno and Teensy 3.0) were used to operate the device. Arduino Uno controlled the sample rotation module (gray dotted box) and the heating/fanning module (pink). For fluorescent detection, Teensy 3.0 generated the excitation waveform (orange) based on Walsh-Hadamard sequency. This wavefunction drove the light-emitting diode (M470D2) module (blue). The emitted light from a sample was detected by a photodiode (S1223) and amplified (green). Teensy 3.0 digitized this signal and performed the inverse Walsh-Hadamard transform to recover the fluorescent intensity.

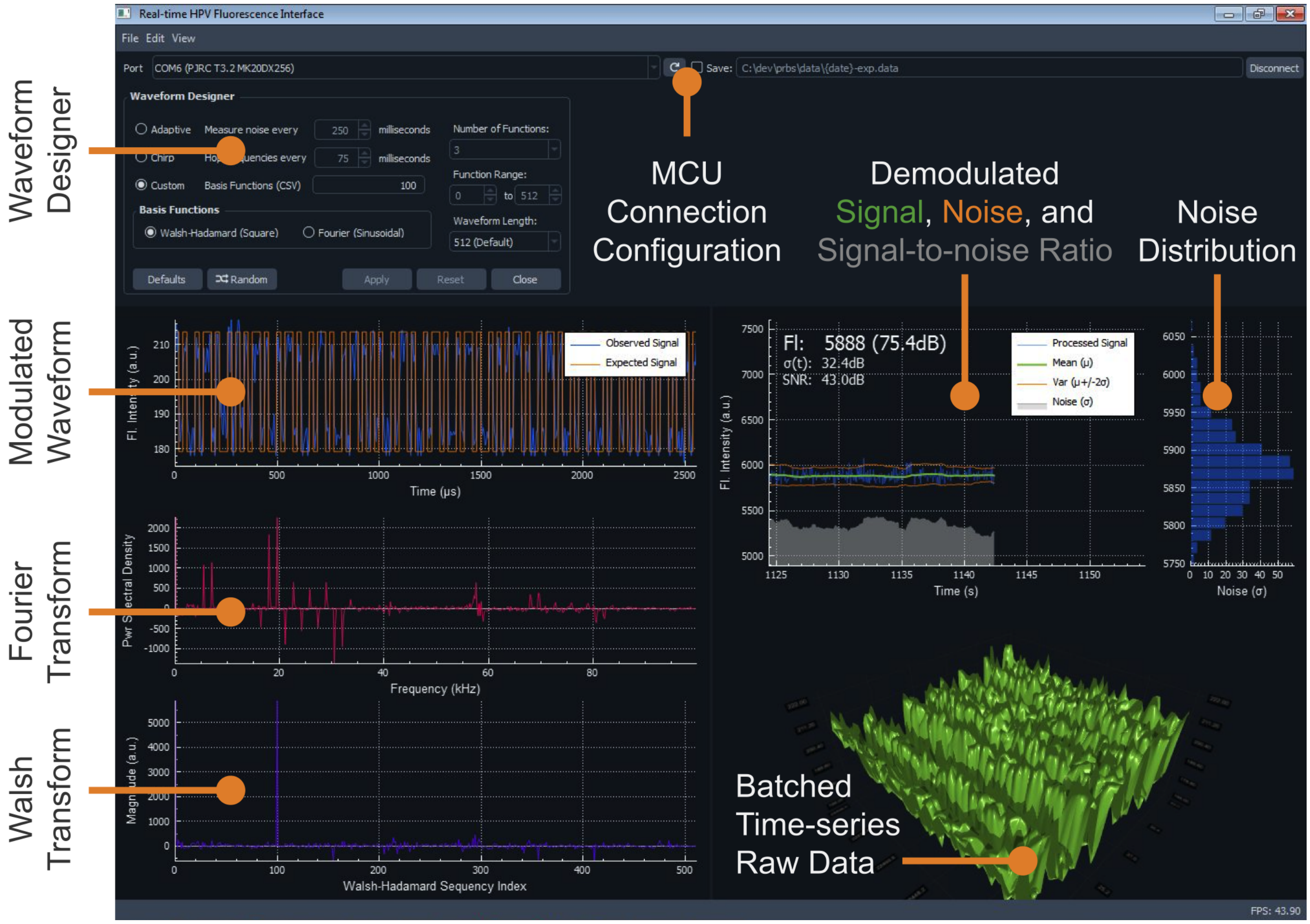

Supplementary Figure 2. CreDiT user interface. The program, running on a computer, presents an extended user interface to set waveforms for signal detection (Waveform Designer). It also receives raw data from the CreDiT microcontroller (MCU) and presents demodulated signal and real-time SNRs. The program was written in C++ using Qt widgets.

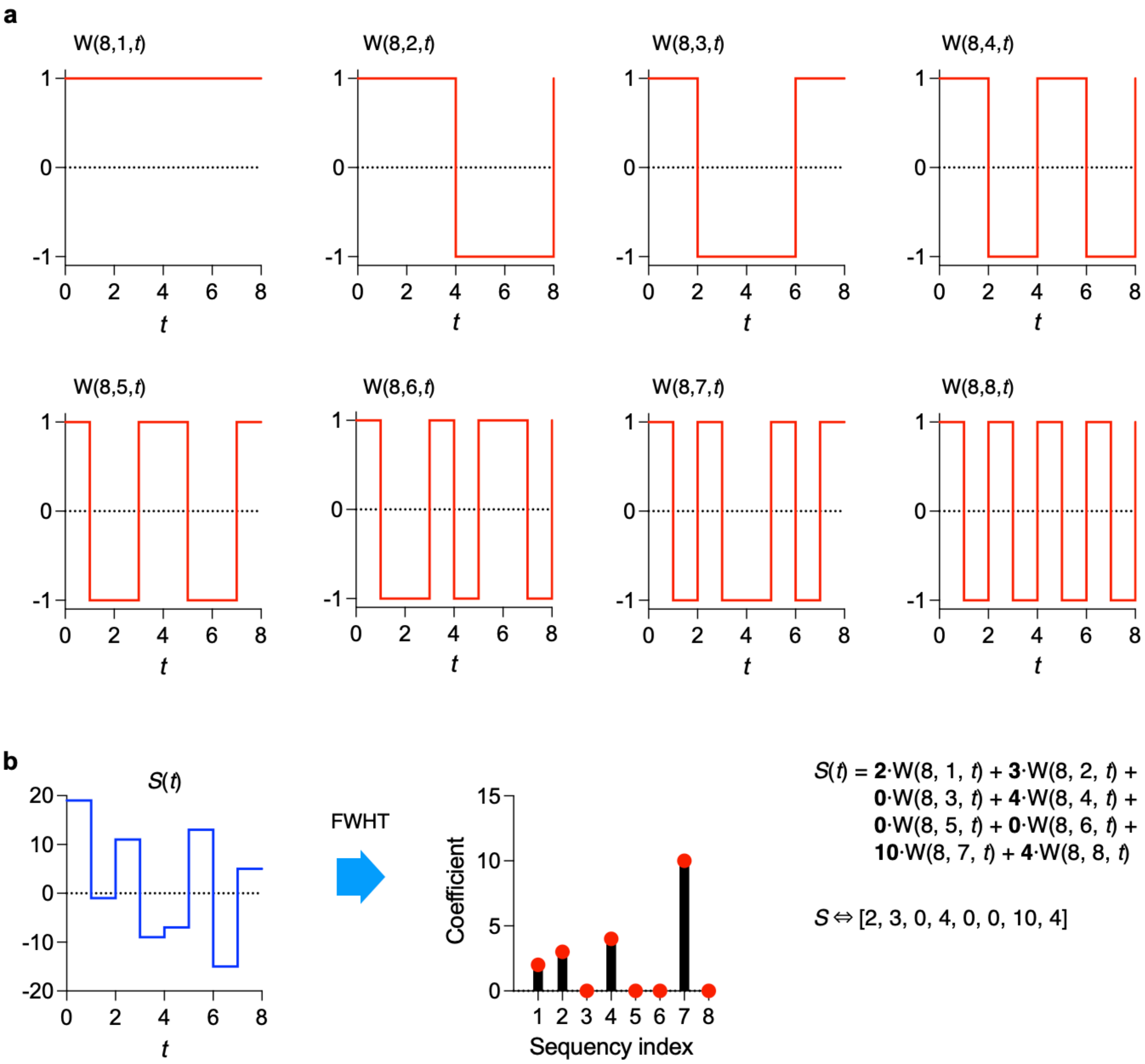

Supplementary Figure 3. Examples of Walsh functions and Walsh-Hadamard analysis. (a) Walsh functions consist of trains of square pulses and have binary values (-1 or 1). The function is denoted as W(*n*, *k*, *t*), where *n* is the function size, *k* is the sequence index, and *t* is the time. Walsh functions with nonidentical sequence indices are orthogonal to each other. Shown are all Walsh functions for *n* = 8. **(b)** A given signal S(*t*) was represented as a weighted sum of Walsh functions (right). The conversion process used the fast Walsh-Hadamard transform (FWHT) algorithm that involves only light computations (addition and subtraction of real numbers). The collection of coefficients [2, 3, 0, 4, 0, 0, 10, 4] can be used to reproduce *S*(t) through the inverse FWHT.

```python
def fwht(a) -> None:
    h=1
    while h < len(a):
        for i in range(0, len(a), h * 2):
            for j in range(i, i + h):
                x = a[j]
                y = a[j + h]
                a[j] = x + y
                a[j + h] = x - y
        h *= 2
```

Supplementary Figure 4. Python code for the fast Walsh-Hadamard transform (FWHT). This code takes the input signal (a) and calculates the coefficient array (h) for Walsh functions. Note that the algorithm involves only additions and subtractions of real numbers, which makes the computation fast and efficient. For the CreDiT device, we implemented a C++ version of this code, optimized for the ARM-Cortex series of microcontrollers. The full code is available at the following GitHub Repository (https://github.com/deganii/fwht-arm).

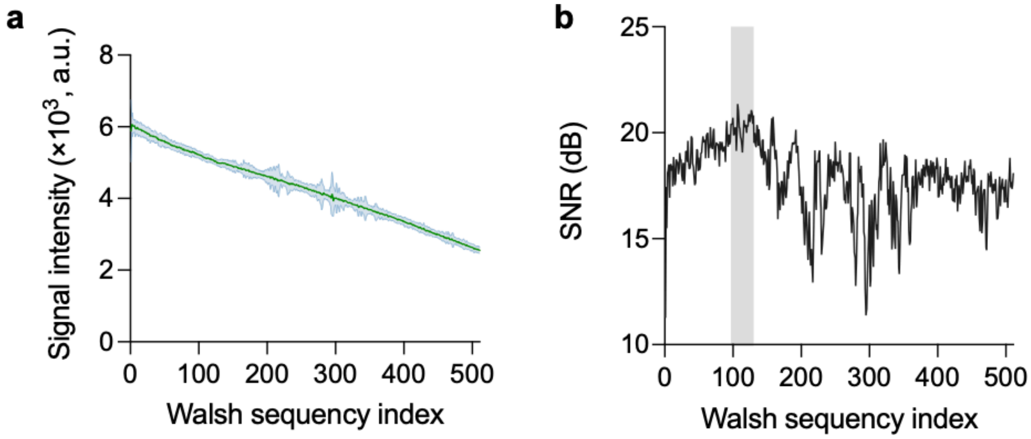

Supplementary Figure 5. Determining the optimal Walsh sequency for excitation signals. (a) Fluorescent intensities were measured using the CreDiT system, while the Walsh sequency was swept from 0 to 512. The mean fluorescence (green) is the average from 60 wavetrains at each sequency. The standard deviation is shown as a 95% confidence interval (blue). **(b)** The signal-to-noise ratio (SNR) was computed based on the measured data. The system achieved high SNR within the Walsh sequency range of 97 and 130 (gray shade). The peak SNR was observed at the sequency 107. a.u., arbitrary unit. Source data are provided as a Source Data file.

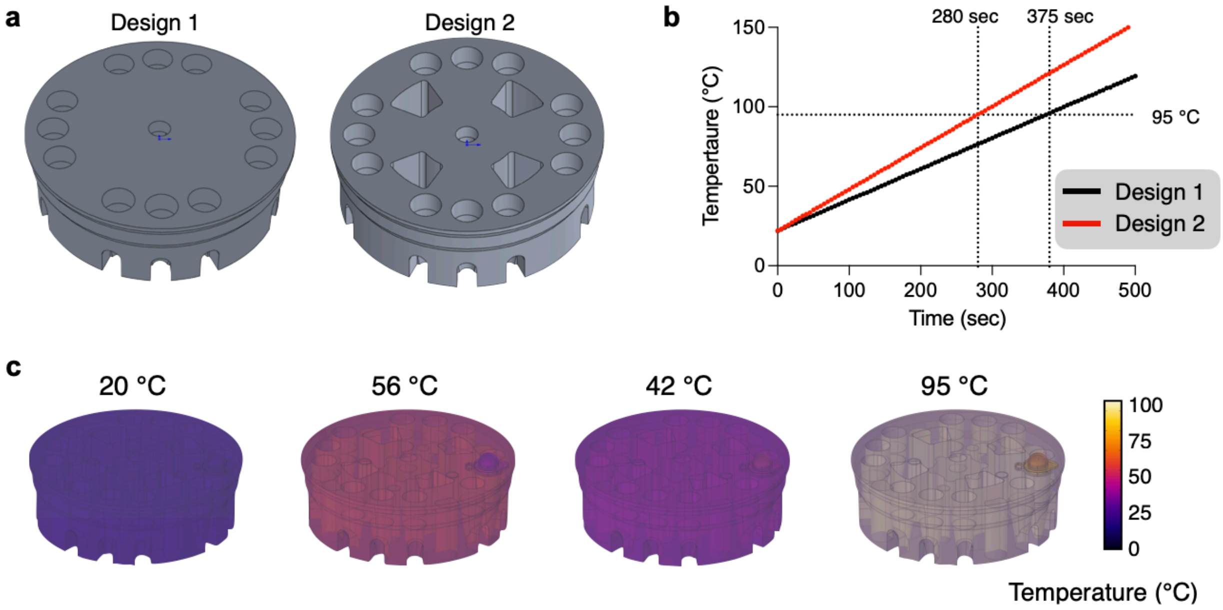

Supplementary Figure 6. Thermal simulation of sample holders. (a) Two candidates (design 1 and design 2) were used for thermal simulations. Each design has mounting holes for 12 samples. The design 1 has a solid body, whereas the design 2 has four pockets to reduce the thermal mass. The heating source was four 15 W heaters embedded in the holder, and the holder material was set to aluminum. **(b)** Simulated heating profile. The temperature at the sample position was estimated. The design 2 (with pockets) showed a faster heating response than the design 1 (without pockets). Source data are provided as a Source Data file. **(c)** Based on the simulation results, the temperature profile of the holder (design 2) was visualized. Note the uniform temperature distribution across the holder.

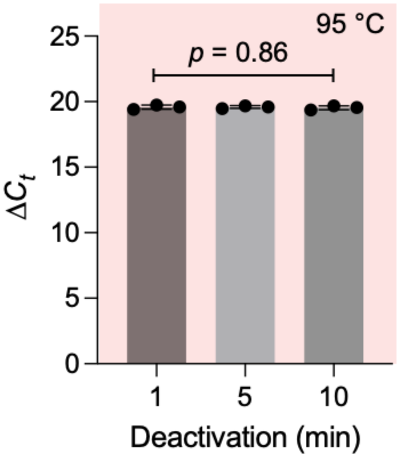

Supplementary Figure 7. Optimization of proteinase K deactivation time in NA extraction. Incubating samples for 1 min at 95 °C was sufficient to deactivate proteinase K (0.5 mg/mL) for downstream analysis. NAs were extracted from Ca Ski cells and subjected to qPCR (500 cells in 25 μL). We calculated $\Delta C_t = C_{t,0} - C_t$, where C_t and $C_{t,0}$ were the threshold cycles from an NA sample and blank, respectively. No significant difference in ΔC_t was observed among samples with different deactivation times ($p = 0.86$; one-way ANOVA). Data are displayed as mean ± s.d. from triplicate ($n = 3$) measurements. Source data are provided as a Source Data file.

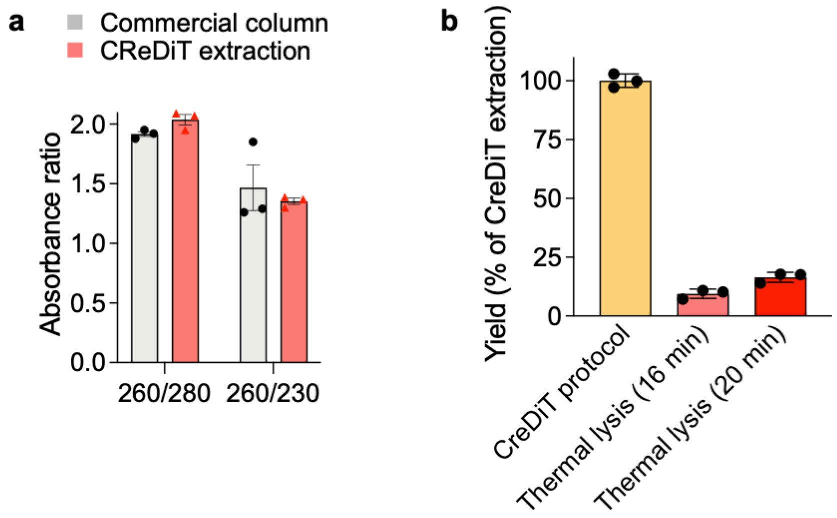

Supplementary Figure 8. Comparison of NA extraction methods. (a) Commercial column vs. CreDiT (Pro K-based) protocol. We measured key metrics of NA sample quality, the absorbance ratio between 260 nm and 280 nm (260/280; purity from proteins) and that between 260 nm and 230 nm (260/230; purity from organic compounds). NA samples extracted by CreDiT had absorbance ratios similar to those prepared by a commercial column. Data are displayed as mean ± s.d. from triplicate (*n* = 3) measurements. **(b)** Thermal lysis vs. CreDiT protocol. For thermal lysis, we heated samples to 95 °C and maintained the temperature for 16 or 20 minutes. Extracted NA was then subjected to qPCR to measure HPV16. The CreDiT protocol led to >6-fold higher signal than thermal lysis, indicating its higher NA-extraction yield. Test samples contained Ca Ski cells (2.5×10^4 cells/mL). HPV16 signals from CreDiT extraction were used for normalization. Data are displayed as mean ± s.d. from technical triplicates (*n* = 3). Source data are provided as a Source Data file.

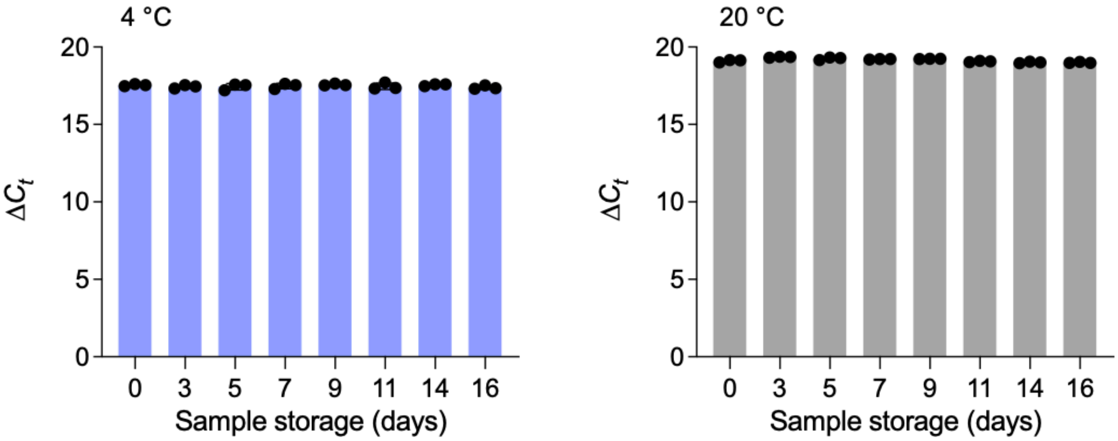

Supplementary Figure 9. Sample storage. Samples containing cervical cancer cells (Ca Ski, 8000 cells) were stored in a refrigerator (4 °C; left) and in an ambient condition (20 °C; right). Following the designated storage time, NAs were extracted from the cells and quantified by qPCR. The ΔC_t values were calculated as $\Delta C_t = C_{t,0} - C_t$ where C_t and $C_{t,0}$ were the threshold cycles from an NA sample and blank, respectively. For both storage conditions, ΔC_t values remained similar to their initial values at day 0 (fresh sample). Data are displayed as mean ± s.d. from technical triplicates (n = 3). Source data are provided as a Source Data file.

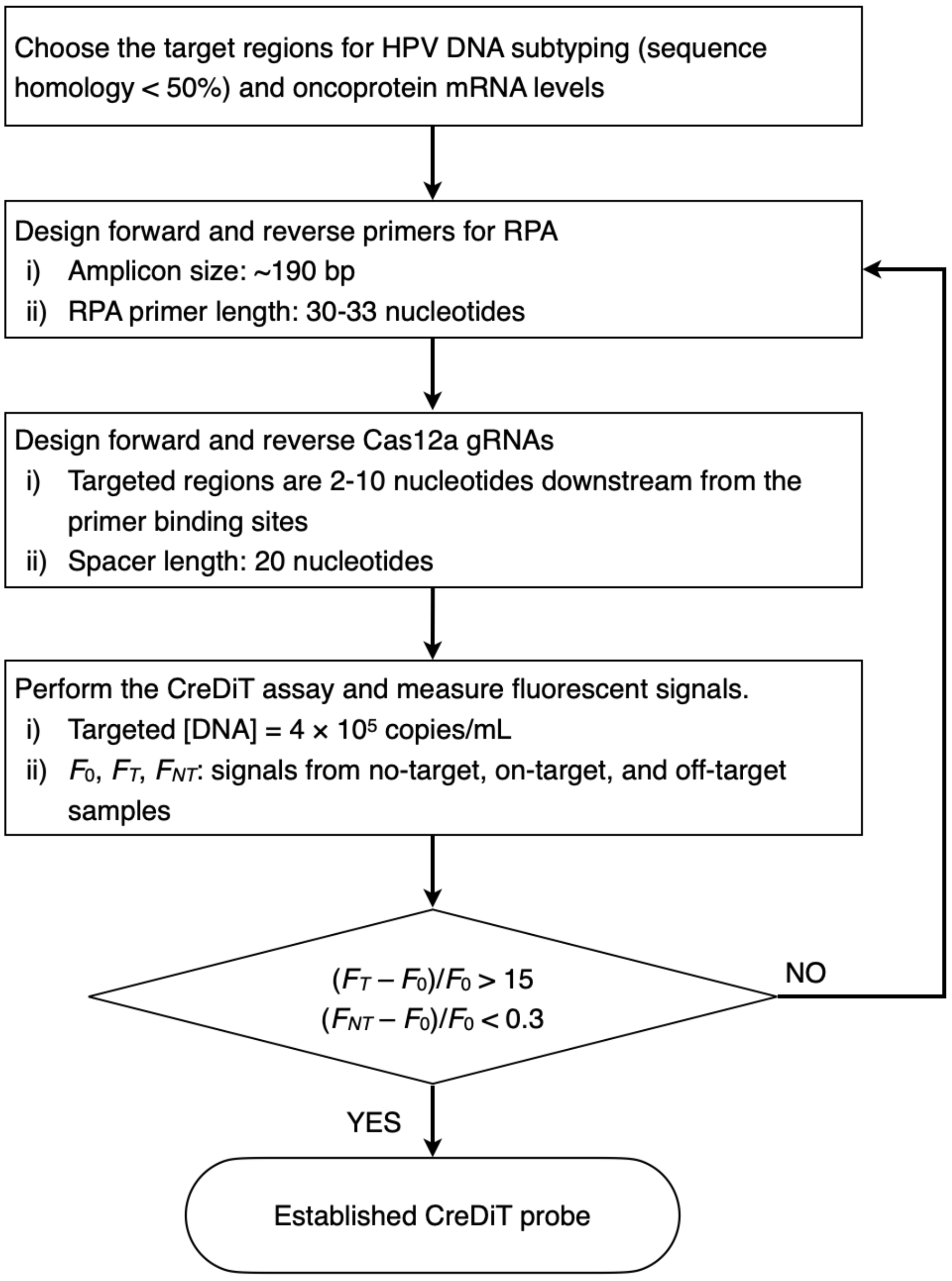

Supplementary Figure 10. Flowchart for designing CreDiT probes.

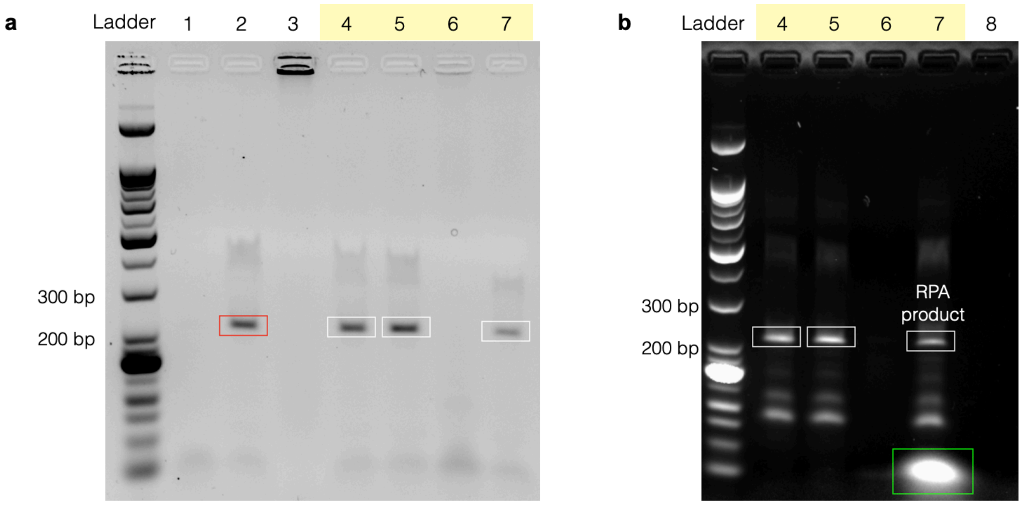

Initial target DNA	−	+	−	+	+	−	+
RPA primers	+	+	−	+	+	+	+
RPA agents	+	+	−	+	+	+	+
Cas12a	−	−	+	+	−	+	+
gRNA	−	−	+	−	+	+	+
F-Q probe	−	−	−	+	+	+	+
	1	2	3	4	5	6	7

Initial target DNA	+	+	−	+	−
RPA primers	+	+	+	+	−
RPA agents	+	+	+	+	−
Cas12a	+	−	+	+	−
gRNA	−	+	+	+	−
F-Q probe	+	+	+	+	+
	4	5	6	7	8

Supplementary Figure 11. Gel electrophoresis analysis on the CreDiT assay products. (a) HPV16 DNA was used as a model target with a concentration of 4 × 10^7 copies/mL. Lanes 1 and 2 were RPA controls, confirming target-specific DNA amplification (red rectangle). Lane 3 was the control for Cas12a/gRNA with excess amounts of Cas12a (5.12 µM) and gRNA (1.28 µM) applied. Lanes 4 and 5 had the RPA reaction conditions in the presence of Cas12a (640 nM) or gRNA (160 nM), respectively. Lanes 6 and 7 had the complete CreDiT reaction condition without (Lane 6) or with (Lane 7) the target DNA input. The RPA reactions successfully produced DNA amplicons (white rectangles) regardless of the presence of Cas12a, gRNA, or both. **(b)** For the assay conditions in Lanes 4 to 7 in (a), we further confirmed the cleavage of reporter probes, i.e., single-stranded DNAs tagged with a fluorescent dye (F) and quencher (Q) pair. Only Lane 7, which had the complete CreDiT reaction in the presence of the target DNA, showed the fluorescent signal (green rectangle) from the cleaved reporters. Lane 8 was from a control sample containing F-Q DNA probes only. The gel was stained for nucleic acids with GelRed® (Biotium). Two separate fluorescent images were acquired from the same gel, detecting GelRed and the reporter fluorophore (fluorescein amidite). The two images were then merged; whole gel images are shown in **Supplementary Fig. 20**. Experiments were performed twice, and representative images were shown. bp, base pairs.

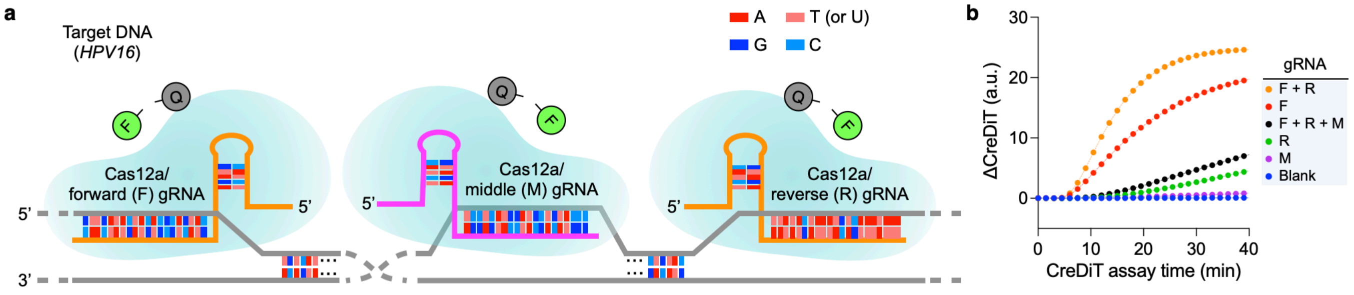

Supplementary Figure 12. Impact of gRNA combinations on CreDiT assay signals. (a) Three gRNAs were designed to target distinct regions of the HPV16 DNA: forward (F), reverse (R), and middle (M). **(b)** The CreDiT assay was performed using various combinations of these gRNAs. The combination of F-gRNA and R-gRNA (the CreDiT protocol) yielded the strongest analytical signal. Notably, this combined signal was close to the sum of the signals obtained using individual gRNAs. Incorporating the M-gRNA, which displayed the weakest signal as an individual gRNA, resulted in a lower overall CreDiT signal. Because the total gRNA amount was fixed (480 nM), adding the less efficient M-gRNA likely reduces the availability of F-gRNA and R-gRNA for Cas12a binding. [HPV16 DNA] = 4 × 10^7 copies/mL. ΔCreDiT is the background-subtracted signal. Data are displayed as mean from technical triplicates (n = 3). a.u., arbitrary unit. Source data are provided as a Source Data file.

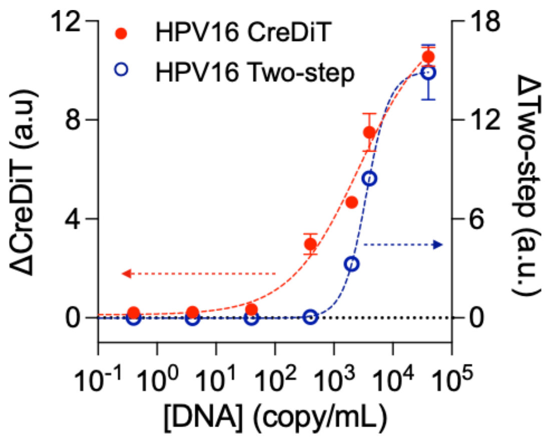

Supplementary Figure 13. Comparison between CreDiT and a two-step assay. Serially diluted HPV16 target DNA samples were analyzed by CreDiT (20 min) and a two-step assay in which RPA (20 min) was followed by Cas12a reaction (30 min). To detect double-stranded DNA (i.e., RPA products), the two-step assay used a gRNA specific to the HPV16 target DNA sequence adjacent to the PAM site. CreDiT exhibited a lower detection limit (40 copies/mL) and a wider dynamic range (4 orders of magnitude) than the two-step assay (detection limit, 400 copies/mL; dynamic range, 2.5 orders of magnitude). ΔCreDiT and ΔTwo-step are the background-subtracted signals. Data are displayed as mean ± s.d. from technical triplicates (n = 3). a.u., arbitrary unit. Source data are provided as a Source Data file.

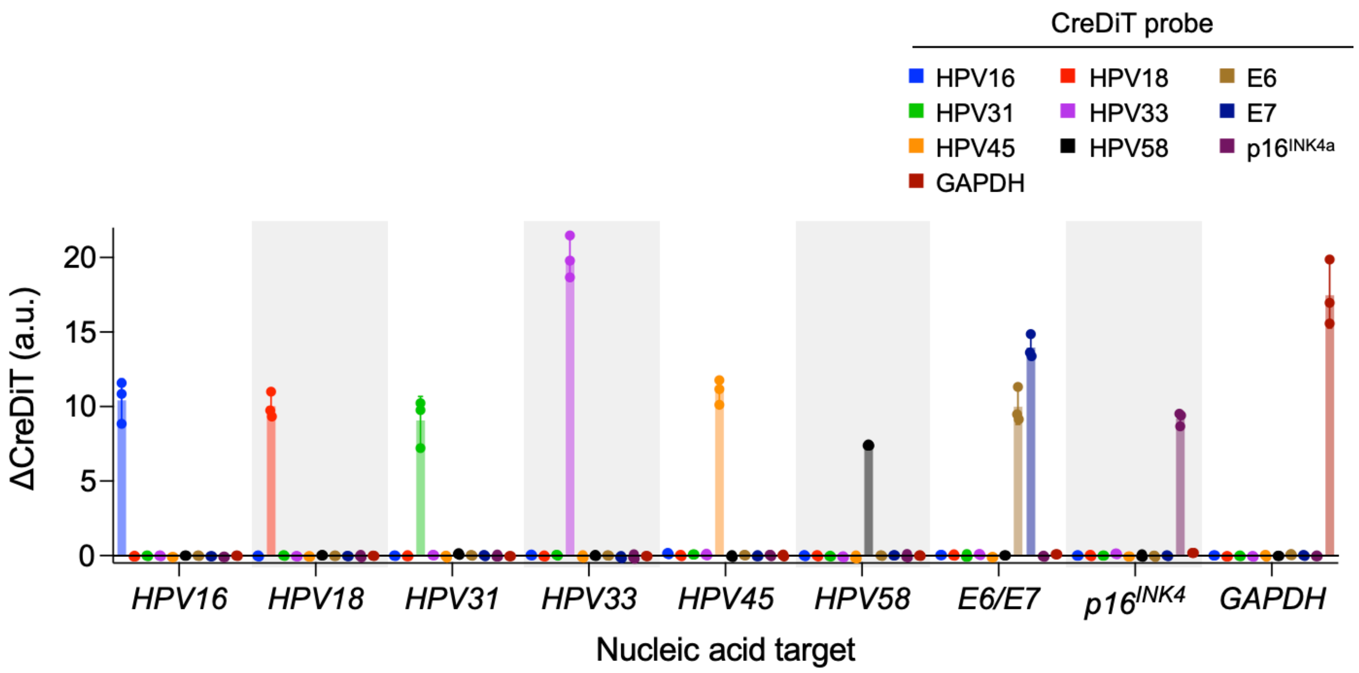

Supplementary Figure 14. Selectivity of CreDiT probes. CreDiT probes were tested using samples containing a single type of NA target (4×10^7 copies/mL). Signals from off-target samples were negligible compared to those from on-target samples. Data are displayed as mean ± s.d. from triplicate ($n = 3$) measurements. a.u., arbitrary unit. Source data are provided as a Source Data file.

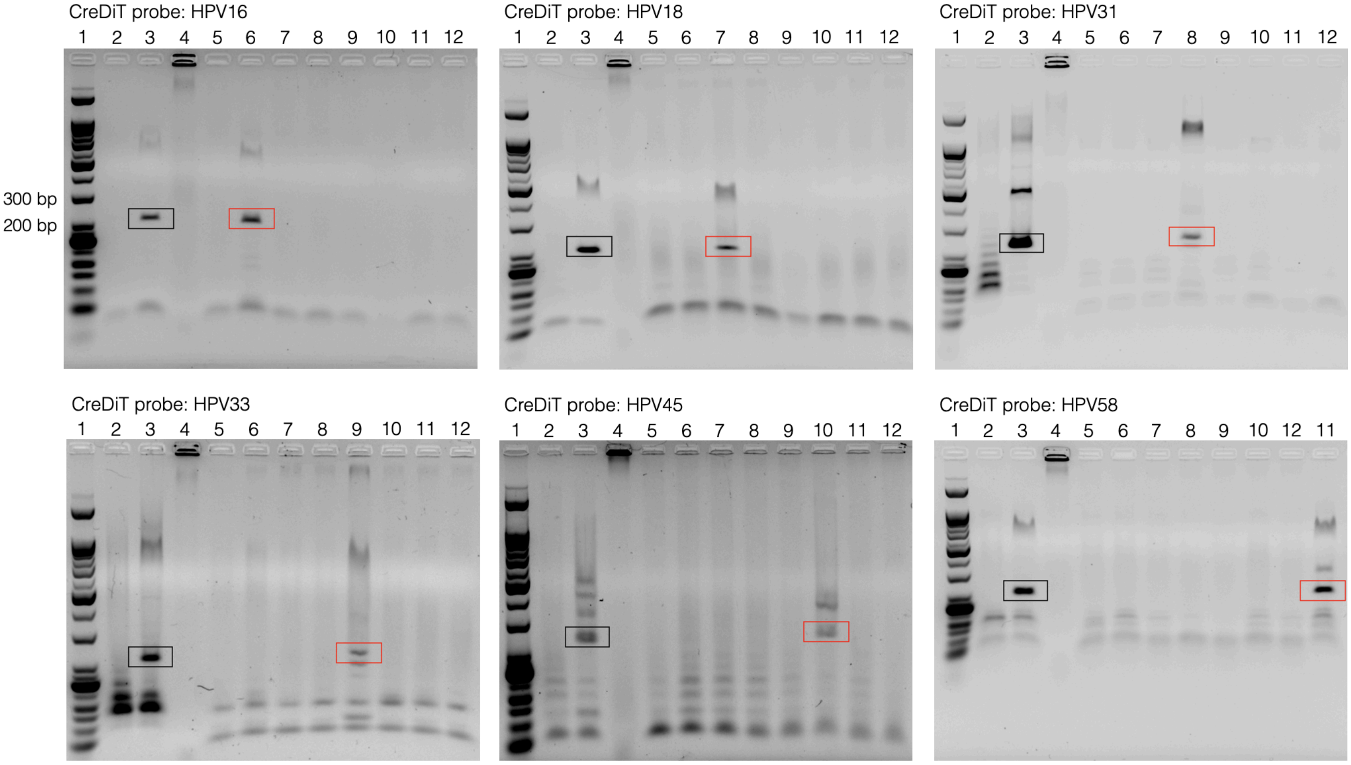

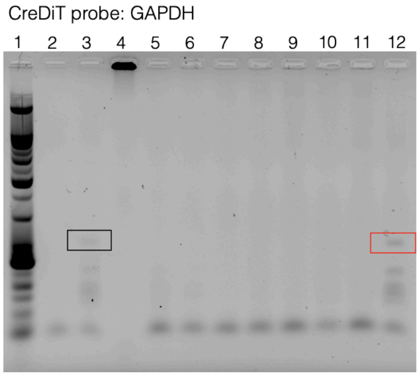

Lane	Experiment condition	
1	Molecular ladder	
2	RPA negative control	Target-specific RPA reagent only
3	RPA positive control	Target-specific RPA reagent + Target DNA
4	Cas12a and gRNA only control	Cas12a (5.12 μM) + gRNAs (1.28 μM)
5	CreDiT reagent only control	Target-specific CreDiT probe† only
6	Input DNA - *HPV16*	
7	Input DNA - *HPV18*	
8	Input DNA - *HPV31*	
9	Input DNA - *HPV33*	Target-specific CreDiT probe† + Input DNA
10	Input DNA - *HPV45*	
11	Input DNA - *HPV58*	
12	Input DNA - *GAPDH*	

† Cas12a (640 nM), gRNAs (60 nM), RPA primers (240 nM)

Supplementary Figure 15. Probe specificity. CreDiT assays were performed using probes designed to detect various hrHPV targets. The corresponding electrophoretic results show that target DNA (red rectangles) was amplified only when its matching probes were present in the CreDiT. The results confirmed the high specificity of the CreDiT assay. Lane 3 is the positive control wherein the target DNA was amplified via RPA only; amplicons are indicated by black rectangles. The initial concentration of an input DNA was 4 × 10^7 copies/mL for all reactions. The whole gel images are shown in **Supplementary Fig. 21**. Experiments were performed twice, and representative images were shown. bp, base pairs.

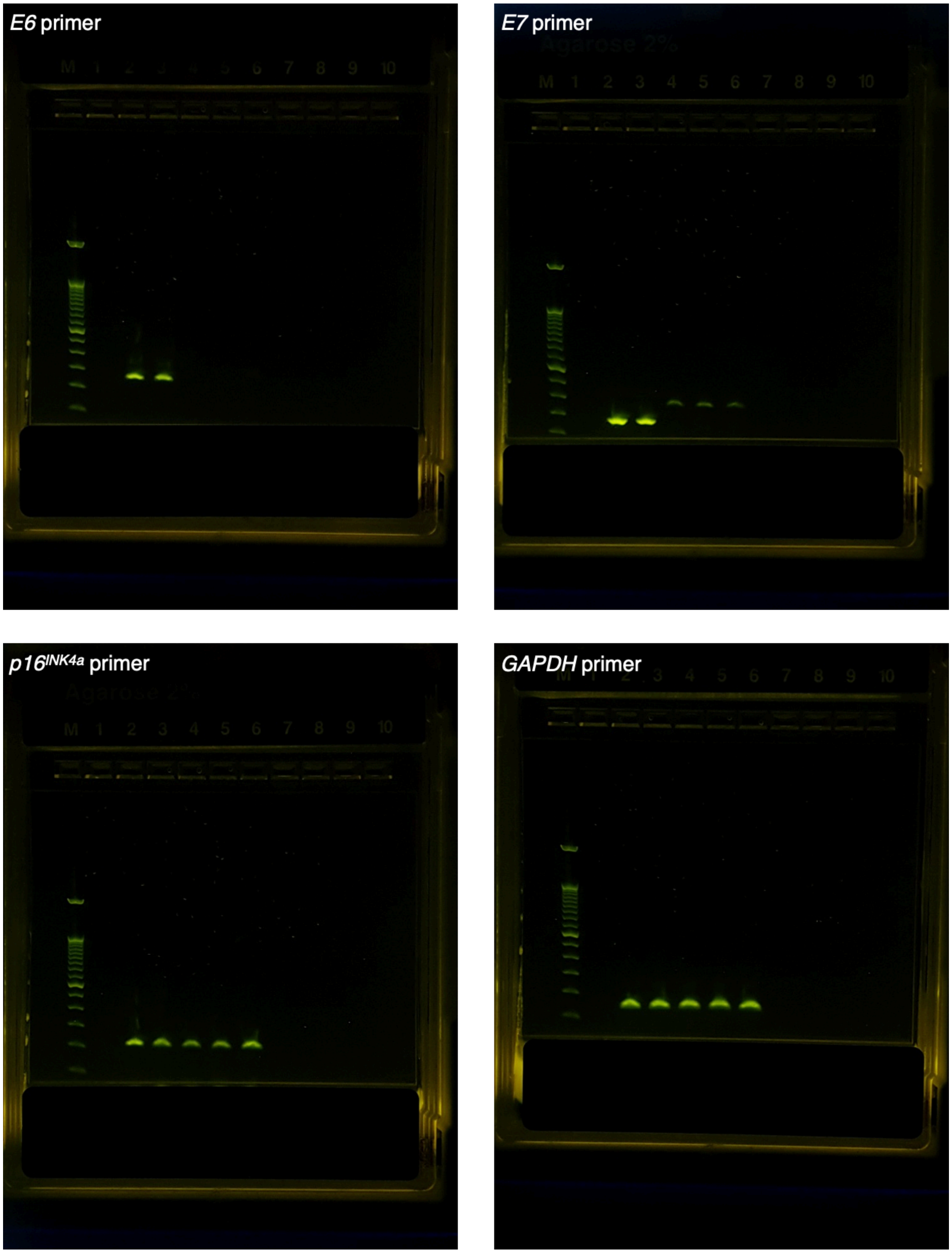

Supplementary Figure 16. Whole gel images for Fig. 5f in the main text. From left to right, the lanes are DNA ladder and RT-PCR products for blank control and RNA extracted from Ca Ski, SiHa, HeLa, C-33 A, and TIOSE6.

a

Usage	Target	Probe[a]	Sequence (5’ to 3’)
RT-PCR for mRNA level analysis	E6[a]	Forward primer	ACC CAG AAA GTT ACC ACA GTT AT
		Reverse primer	TTT ATC ACA TAC AGC ATA TGG ATT CC
	E7[a]	Forward primer	CAA CCA GAG ACA ACT GAT CTC
		Reverse primer	GAA TGT CTA CGT GTG TGC TTT
	p16^{INK4a}	Forward primer	GCA TAG ATG CCG CGG A
		Reverse primer	ATG AAA ACT ACG AAA GCG GG
	GAPDH	Forward primer	ACC AGG GCT GCT TTT AAC
		Reverse primer	GTG CCA TGG AAT TTG CCA T

[a] The primers were designed to target mRNA of E6 and E7 of HPV16

b

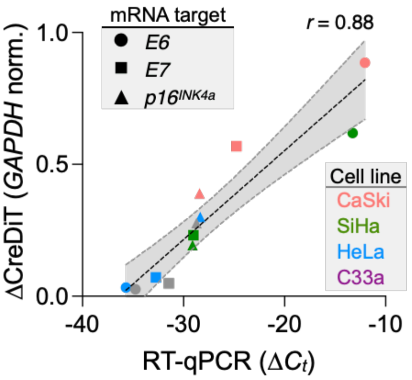

Supplementary Figure 17. RT-qPCR for mRNA of HPV-associated oncoproteins in cell lines. (a) Primer sequences for RT-qPCR. **(b)** Profiling of mRNA levels of HPV-associated oncoproteins in cervical cancer cell lines. The CreDiT results (**Fig. 5e** in the main text) showed a good concordance with those of RT-qPCR. *r*, Pearson’s coefficient. Each data point represents a mean value from technical triplicate samples containing 4×10^5 cells/mL. Source data are provided as a Source Data file.

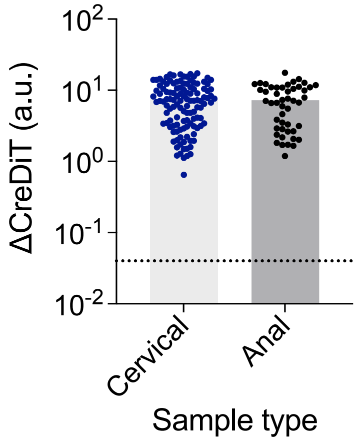

Supplementary Figure 18. ***GAPDH*** **signals in clinical samples.** The signal values by CreDiT were above the LOQ cutoff (dotted line) for all cervical brushing (n = 121) and anal swap (n = 48) samples. Each data point is a mean value from triplicate measurements. The bar represents a mean value. a.u., arbitrary unit. Source data are provided as a Source Data file.

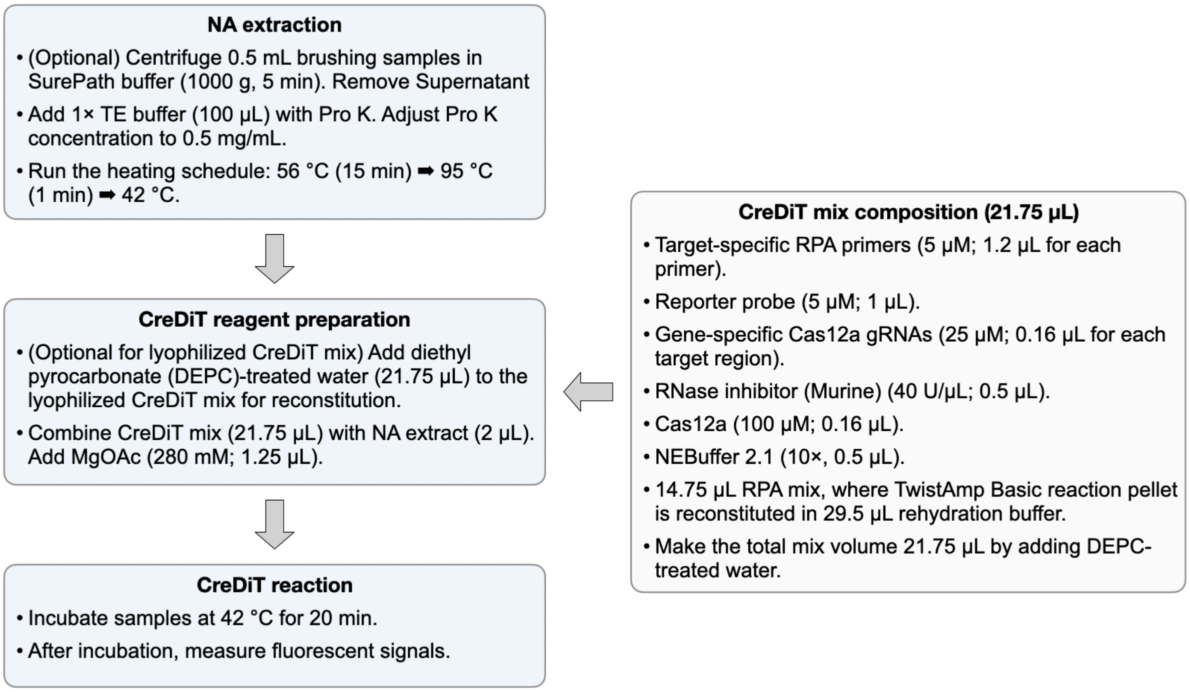

Supplementary Figure 19. CreDiT workflow.

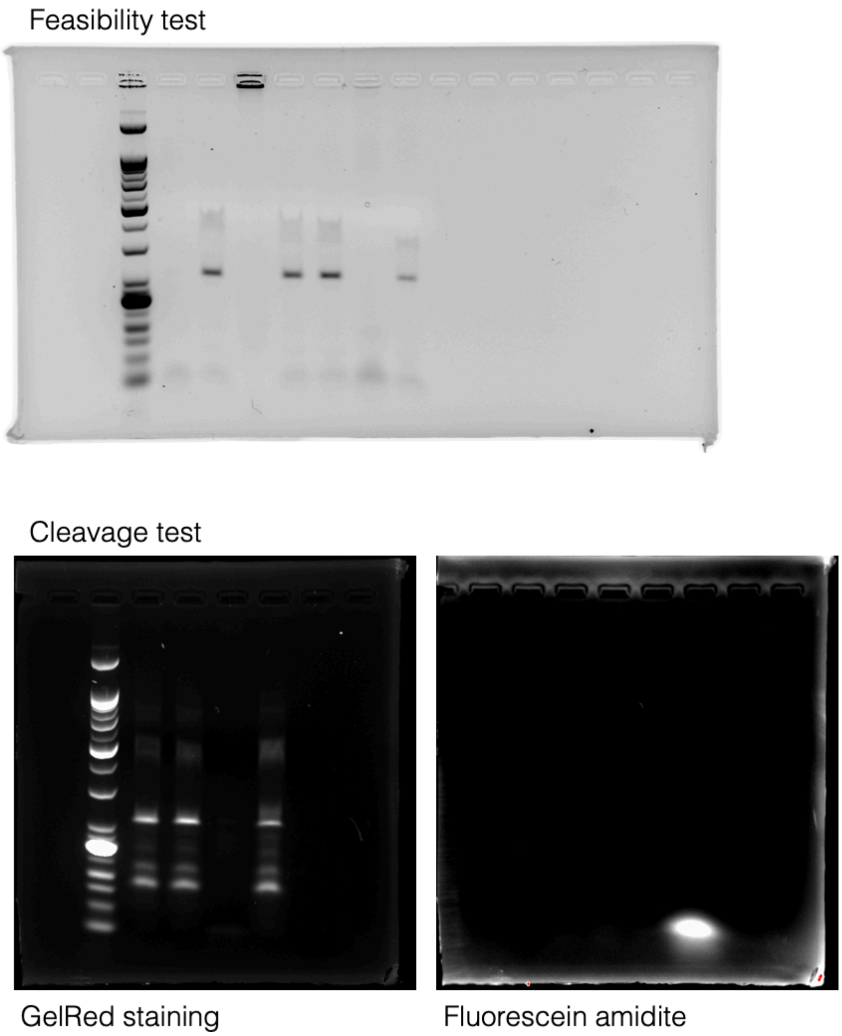

Supplementary Figure 20. Whole gel images for Supplementary Fig. 11.

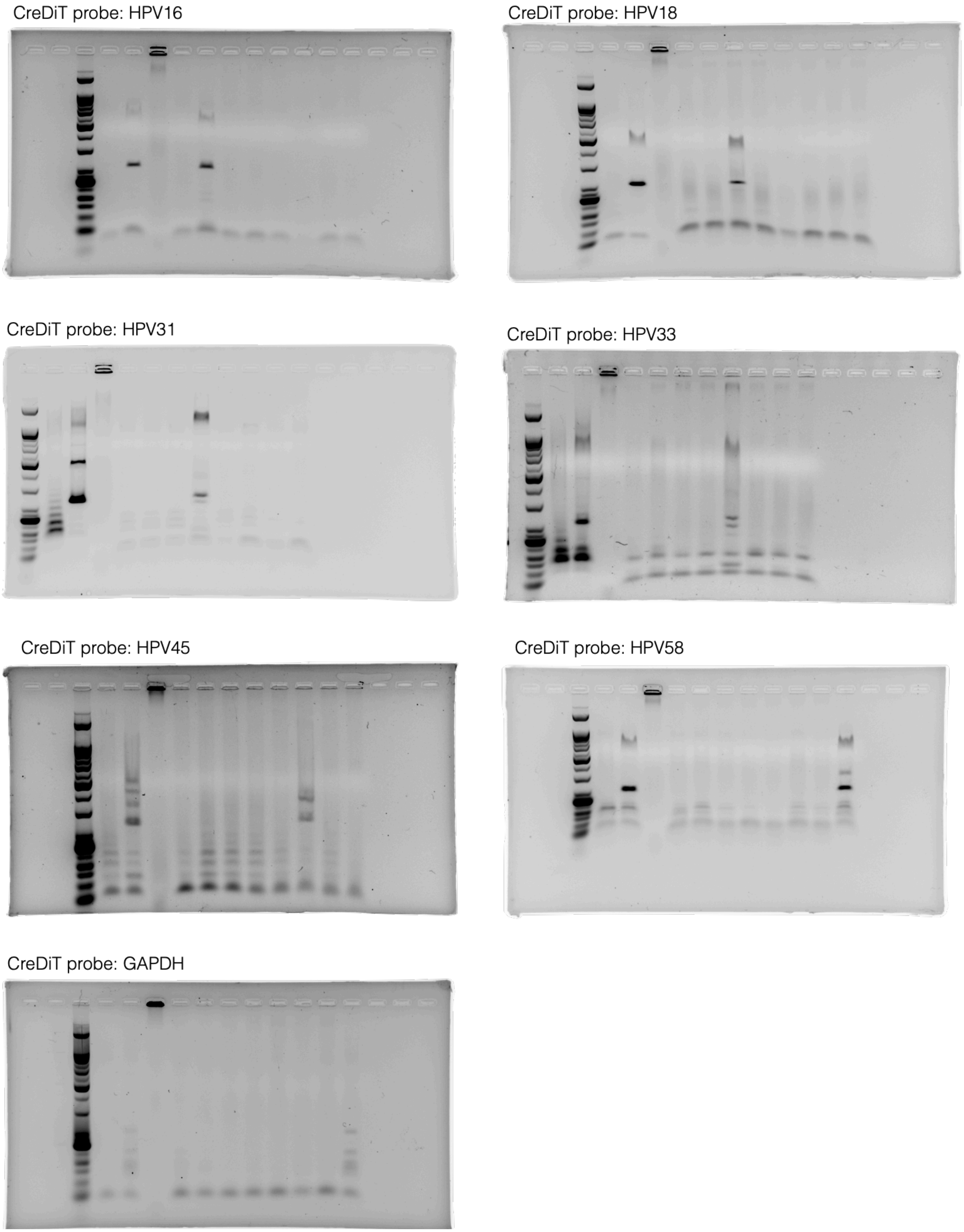

Supplementary Figure 21. Whole gel images for Supplementary Fig. 15.

Supplementary Table 1. Comparison with CRISPR-based HPV molecular tests.

System	Readout	NA detection time (min)†	LOD	Assay characteristics	Reference
Electrochemically active electrode	Electrochemistry	70	50 pM	• Specialized chip fabrication<br>• Low sensitivity<br>• Narrow target coverage<br>• Absence of clinical study	1
Electrochemically active electrode	Electrochemistry	60	30 pM	• Specialized chip fabrication<br>• Low sensitivity<br>• Narrow target coverage<br>• Absence of clinical study	2
Electrochemiluminiscence-active electrode	Electrochemiluminesecence	70	0.48 pM	• Specialized chip fabrication<br>• Low sensitivity<br>• Narrow target coverage<br>• Absence of clinical study	3
Lateral flow assay; RPA+Cas	Color	>180	0.24 fM	• Need for several steps and reagent<br>• Subjective, qualitative results<br>• Narrow target coverage<br>• Long assay time	4
Lateral flow assay; LAMP+Cas	Color	60	N/A	• Need for several steps and reagent<br>• Subjective, qualitative results<br>• Complicated probe design<br>• Narrow target coverage	5
Lateral flow assay; RPA+Cas	Color	105	3.3 aM	• Specialized chip fabrication<br>• Need for several steps and reagents<br>• Need for a specialized reporter<br>• Complicated probe design<br>• Narrow target coverage	6
Lateral flow assay; RPA+Cas	Color	30	1 aM	• Specialized chip fabrication<br>• Need for several steps and reagents<br>• Subjective, qualitative results<br>• Narrow target coverage	7
Dynamic aqueous multiphase reaction; RPA+Cas	Fluorescence	60	5 aM	• Narrow target coverage	8
CDetection; RPA+Cas	Fluorescence	190	1 aM	• Involving two steps<br>• Narrow target coverage<br>• Long assay time	9
DETECTR; RPA+Cas	Fluorescence	70	Attomolar	• Involving two steps<br>• Narrow target coverage	10
Polydisperse droplet digital assay	Fluorescence	30	100 aM	• Narrow target coverage	11
Microfluidic dual-droplet device; RPA+Cas	Fluorescence	30	1 aM	• Specialized chip fabrication and signal measurement (i.e. imaging)<br>• Delicate care of droplets<br>• Narrow target coverage	12
MiCaR; RPA+Cas	Fluorescence	>40	1.7 fM	• Specialized chip fabrication and signal measurement (i.e. imaging)<br>• Complicated assay preparation<br>• 2 steps	13
DROPT; RPA+Cas	Fluorescence	30	1 aM	• Narrow target coverage<br>• 2 steps	14
CreDiT (Our work)	Fluorescence	20	66 zM	• Single step<br>• Broad target coverage	-

† This metric only considers the duration for nucleic acid (NA) detection, excluding the time for NA extraction.

Supplementary Table 2. Oligonucleotide sequences used in this study.

Usage	Target	Probe[a]	Sequence (5' to 3')
HPV subtyping	HPV16	Forward RPA primer	CAT TTG TTG GGG TAA CCA ACT ATT TGT TAC T
		Reverse RPA primer	CGT CTG CAG TTA AGG TTA TTT TGC ACA GTT G
		Forward Cas12a gRNA	UAA UUU CUA CUA AGU GUA GAU *GUA CUG CGU GUA GUA UCA AC*
		Reverse Cas12a gRNA	UAA UUU CUA CUA AGU GUA GAU *AAU AUG AUU UAC AGU UUA UUU*
		Extra Cas12a gRNA	UAA UUU CUA CUA AGU GUA GAU *AGG AGU ACC UAC GAC AUG GG*
	HPV18	Forward RPA primer	TTG CTG GCA TAA TCA ATT ATT TGT TAC TGT G
		Reverse RPA primer	GCA GTT AAA GTA ATA GTA CAC AAC TGA AAA AT
		Forward Cas12a gRNA	UAA UUU CUA CUA AGU GUA GAU *AAU UGG UAC UGC GAG UGG UAU*
		Reverse Cas12a gRNA	UAA UUU CUA CUA AGU GUA GAU *CAG ACA UGU UGA GGA AUA UGA*
	HPV31	Forward RPA primer	CCA CAC GTA GTA CCA ATA TGT CTG TTT GTG C
		Reverse RPA primer	CAA TCT TCC AAA ATA GCA GGA TTC ATA CTG T
		Forward Cas12a gRNA	UAA UUU CUA CUA AGU GUA GAU *AGU AUC ACU GUU UGC AAU UG*
		Reverse Cas12a gRNA	UAA UUU CUA CUA AGU GUA GAU *GCA GAC AUA AUG ACA UAU AU*
	HPV33	Forward RPA primer	CCA CTC GCA GTA CTA ATA TGA CTT TAT GCA C
		Reverse RPA primer	CCA ATC TTC TAA AAT ATC TGG ATT CAT AGC A
		Forward Cas12a gRNA	UAA UUU CUA CUA AGU GUA GAU *AUG UAC UGU CAC UAG UUA CU*
		Reverse Cas12a gRNA	UAA UUU CUA CUA AGU GUA GAU *CAG AAG UUA UGA CAU AUA UU*
	HPV45	Forward RPA primer	CAG TGG CTC TAT TAT TAC ATC TGA TTC TCA
		Reverse RPA primer	TTC CTC CAC ATG TCT ACT ATA TTG CTT AAA
		Forward Cas12a gRNA	UAA UUU CUA CUA AGU GUA GAU *GGG CCU UAU GUA ACC AAU AU*
		Reverse Cas12a gRNA	UAA UUU CUA CUA AGU GUA GAU *AAU CCU GUG CCA GGU ACA UA*
	HPV58	Forward RPA primer	TGC ACA AGG TCA TAA CAA TGG CAT TTG CTG
		Reverse RPA primer	GCA AAG CTG AAA AAC AAA CTG TAA GTC ATA T
		Forward Cas12a gRNA	UAA UUU CUA CUA AGU GUA GAU *CGG UAA CAA AUA ACU GAU UG*
		Reverse Cas12a gRNA	UAA UUU CUA CUA AGU GUA GAU *GAA UAU GUA CGU CAU GUU GA*
mRNA level analysis	*E6*[b]	Forward RPA primer	AGG AGC GAC CCA GAA AGT TAC CAC AGT TAT
		Reverse RPA primer	CTT TAA ACA TTT ATC ACA TAC AGC ATA TGG
		Forward Cas12a gRNA	UAA UUU CUA CUA AGU GUA GAU *AUA UCA UGT AUA GUU GUU UG*
		Reverse Cas12a gRNA	UAA UUU CUA CUA AGU GUA GAU *GAU UUA UGC AUA GUA UAU AG*
	E7[b]	Forward RPA primer	GCA ACC AGA GAC AAC TGA TCT CTA CTG TTA T
		Reverse RPA primer	TTC CAA AGT ACG AAT GTC TAC GTG TGT GCT T
		Forward Cas12a gRNA	UAA UUU CUA CUA AGU GUA GAU *UCU GAG CUG UCA UUU AAU TG*
		Reverse Cas12a gRNA	UAA UUU CUA CUA AGU GUA GAU *UCU ACG CUU CGG UUG UGC GU*
	$p16^{INK4a}$	Forward RPA primer	AGT AAC CAT GCC CGC ATA GAT GCC GCG GAA
		Reverse RPA primer	AAA ACT ACG AAA GCG GGG TGG GTT GTG GCG G
		Forward Cas12a gRNA	UAA UUU CUA CUA AGU GUA GAU *UUC AAU CGG GGA UGU CUG AG*
		Reverse Cas12a gRNA	UAA UUU CUA CUA AGU GUA GAU *UCC UAC AGG GCC ACA ACU GC*
Internal positive control	*GAPDH*	Forward RPA primer	ACG GAT TTG GTC GTA TTG GGC GCC TGG TCA C
		Reverse RPA primer	TTG ATG ACA AGC TTC CCG TTC TCA GCC TTG A
		Forward Cas12a gRNA	UAA UUU CUA CUA AGU GUA GAU *CUU UAC CAG AGU UAA AAG CA*
		Reverse Cas12a gRNA	UAA UUU CUA CUA AGU GUA GAU *GAU UCC ACC CAU GGC AAA UU*
		Reporter probe	FAM-TTA TT-BHQ1
PCR	HPV16	Forward primer	TTG TTG GGG TAA CCA ACT
		Reverse primer	GCA GTT AAG GTT ATT TTG CAC AG

[a] Spacer sequences of Cas12a gRNAs are in italics.
[b] The probes were designed to target *E6* and *E7* mRNAs of HPV16.

Supplementary Table 3. CreDiT probe candidates for other DNA and mRNA targets.

Usage	Target	Probe[a]	Sequence (5' to 3')
HPV subtyping	HPV35	Forward RPA primer	TGT GTT CTG CTG TGT CTT CTA GTG ACA GTA C
		Reverse RPA primer	GTG GTG TAA GGC CAA AAT TCC AAT CCT CTA
		Forward Cas12a gRNA	UAA UUU CUA CUA AGU GUA GAU *UUC CUU AAA AUU GUC AUU UU*
		Reverse Cas12a gRNA	UAA UUU CUA CUA AGU GUA GAU *UCA UAG UAU GAA CCC GUC CA*
	HPV39	Forward RPA primer	TCT TAC TGT AGT GGA CAC TAC CCG TAG TAC
		Reverse RPA primer	ATA GTG TGA ATA TAA GAC ATA ACA TCA GTT
		Forward Cas12a gRNA	UAA UUU CUA CUA AGU GUA GAU *UAG AGG UAG AUA AUG UAA AG*
		Reverse Cas12a gRNA	UAA UUU CUA CUA AGU GUA GAU *CAA CUG UGU ACU GUC ACA UU*
	HPV51	Forward RPA primer	ATA CTA CCA GAA GTA CAA ATT TAA CTA TTA
		Reverse RPA primer	AAG AAT GGT AGG ATC CAT TGT GTG TAA ATA A
		Forward Cas12a gRNA	UAA UUU CUA CUA AGU GUA GAU *GGA AAC CGC AGC AGU GGC AG*
		Reverse Cas12a gRNA	UAA UUU CUA CUA AGU GUA GAU *UAC UUU AAC UAC AGA GGU AA*
	HPV52	Forward RPA primer	TCC CAA TTA TTT AAT AAA CCG TAC TGG TTA
		Reverse RPA primer	CCT CGC CAT GAC GAA GGT ATT CCT TAA AAT T
		Forward Cas12a gRNA	UAA UUU CUA CUA AGU GUA GAU *UUA UUG UGG CCC UGC GCA CG*
		Reverse Cas12a gRNA	UAA UUU CUA CUA AGU GUA GAU *GUG AAA AAG GAA AGC ACA UA*
	HPV56	Forward RPA primer	ATG AAT TAC AAT TTG TTT TTC AAT TAT GCA
		Reverse RPA primer	TTG GTG GCT GTT CCC GTT GAC ATG TTA TAG
		Forward Cas12a gRNA	UAA UUU CUA CUA AGU GUA GAU *AAC CUC UGC AGA CAA AGU AA*
		Reverse Cas12a gRNA	UAA UUU CUA CUA AGU GUA GAU *UAU AGA UAU GUU AGA AGC AC*
	HPV59	Forward RPA primer	TAC TCG CAG CAC CAA TCT TTC TGT GTG TGC
		Reverse RPA primer	TCC AAA ATA GTG GTA TTC ATA TTA TGA ATG
		Forward Cas12a gRNA	UAA UUU CUA CUA AGU GUA GAU *TAG GAA TAG AAG ACG TAG TA*
		Reverse Cas12a gRNA	UAA UUU CUA CUA AGU GUA GAU *ACA TTA ACT ACA GAG GTA AT*
	HPV66	Forward RPA primer	CAG AAG CAC CAA CAT GAC TAT TAA TGC AGC
		Reverse RPA primer	TCC AAT CGT CTA ATA AAG TAT TAT TCA TAT
		Forward Cas12a gRNA	UAA UUU CUA CUA AGU GUA GAU *UCA UAU UUA GUU AAU GUG CU*
		Reverse Cas12a gRNA	UAA UUU CUA CUA AGU GUA GAU *GAA GUU AUG GCA UAU UUG CA*
	HPV68	Forward RPA primer	ACC ACT CGC AGT ACT AAT TTT ACT TTG TCT A
		Reverse RPA primer	ATA GCA GGA TTC ATA GTA TGT ATA TAG GAC
		Forward Cas12a gRNA	UAA UUU CUA CUA AGU GUA GAU *CUG GUA CAG CUG AUU CAG UA*
		Reverse Cas12a gRNA	UAA UUU CUA CUA AGU GUA GAU *UAU AAC AUU GUC AAC UGA UG*
mRNA level test	*E6*[b]	Forward RPA primer	CTG TGC ACG GAA CTG AAC ACT TCA CTG CAA
		Reverse RPA primer	TAA TGT CTT AAT TCT CTA ATT CTA GAA TAA
		Forward Cas12a gRNA	UAA UUU CUA CUA AGU GUA GAU *CUU GCA AUA UAC ACA GGU UA*
		Reverse Cas12a gRNA	UAA UUU CUA CUA AGU GUA GAU *UGC AUG CCA UAA AUG UAU AG*
	E7[b]	Forward RPA primer	ATT CCG GTT GAC CTT CTA TGT CAC GAG CAA
		Reverse RPA primer	GCT CGA AGG TCG TCT GCT GAG CTT TCT ACT
		Forward Cas12a gRNA	UAA UUU CUA CUA AGU GUA GAU *CGU UUU CUU CCU CUG AGU CG*
		Reverse Cas12a gRNA	UAA UUU CUA CUA AGU GUA GAU *UGU GAA GCC AGA AUU GAG CU*

[a] Spacer sequences of Cas12a gRNAs are in italics.
[b] The probes were designed to target *E6* and *E7* mRNAs of HPV18.

Supplementary Table 4. CreDiT diagnostic statistics with cervical/vaginal brushing samples.

Targets	Positive vs. negative			
	Area under the curve	Sensitivity	Specificity	Accuracy
HPV16	0.992	1.000	0.917	0.975
HPV18	0.996	1.000	0.933	0.992
HPV45	0.997	0.990	0.850	0.967

Supplementary Table 5. Demographic and clinical information of the anal swab samples.

	Anal brushing		Total
**Case**	Negative	HPV	
	16	32	48
**Age**			
Median	48	53	53
Range	26-76	32-77	26-77
**Gender**			
Male	12 (75%)	23 (72%)	35 (73%)
Female	4 (25%)	9 (28%)	13 (27%)
**Subtype**			
HPV16	-	26 (81%)	26 (54%)
HPV18	-	5 (16%)	5 (10%)
HPV45	-	9 (28%)	9 (19%)
Others†	-	17 (53%)	17 (35%)

†This category combines hrHPV subtypes of HPV31, 33, 35, 39, 51, 52, 56, 58, 59, 66, and 68.

SUPPLEMENTARY NOTE

List of symbols

	Description	Unit
D	concentration of DNA target	M
d	concentration of cleaved DNA target	M
**Cas12a trans-cleavage**		
C	concentration of Cas12a	M
C_a	concentration of Cas12a and substrate complex	M
C_e	concentration of Cas12a and substrate complex after *cis*-cleavage	M
C_0	initial concentration of Cas12a	M
k_{1c}	forward reaction constant for Cas12a *cis*-cleavage	$s^{-1}\cdot M^{-1}$
k_{2c}	backward reaction constant for Cas12a *cis*-cleavage	s^{-1}
k_c	catalytic turnover rate for Cas12a *cis*-cleavage	s^{-1}
K_{Mc}	Michaelis-Menten constant for Cas12a *cis*-cleavage	M
k_{1t}	forward reaction constant for Cas12a *trans*-cleavage	$s^{-1}\cdot M^{-1}$
k_{2t}	backward reaction constant for Cas12a *trans*-cleavage	s^{-1}
k_t	catalytic turnover rate for Cas12a *trans*-cleavage	s^{-1}
K_{Mt}	Michaelis-Menten constant for Cas12a *trans*-cleavage	M
**Polymerase reaction**		
P	concentration of polymerase	M
P_a	concentration of polymerase and substrate complex	M
P_0	initial concentration of polymerase	M
k_{1p}	forward reaction constant for polymerase	$s^{-1}\cdot M^{-1}$
k_{2p}	backward reaction constant for polymerase	s^{-1}
k_p	catalytic turnover rate for polymerase	s^{-1}
K_{Mp}	Michaelis-Menten constant for polymerase	M

The CreDiT assay relies on two concurrent reactions: i) target DNA amplification by RPA and ii) cleavage of signal probes by Cas12a. However, Cas12a activity can impede RPA because activated Cas12a enzyme can degrade both its DNA target (*cis*-cleavage) and RPA primers (*trans*-cleavage). Interestingly, our CreDiT results suggest that RPA outpaces the cleavage reactions, enabling efficient DNA replication within a single reaction mixture. To support this observation, we estimated and compared the reaction rates of these three processes: DNA replication, *cis*-, and *trans*-cleavages.

1. DNA replication and *cis*-cleavage

We consider two simplified reactions: i) a polymerase reaction that amplifies DNA and iii) a single turnaround DNA cleavage by Cas12a. The governing reactions are written as

Cas12a *cis*-cleavage $$C + D \underset{k_{2c}}{\overset{k_{1c}}{\rightleftharpoons}} C_a \xrightarrow{k_c} C_e + d$$

DNA replication $$P + D \underset{k_{2p}}{\overset{k_{1p}}{\rightleftharpoons}} P_a \xrightarrow{k_p} P + D + D$$

These two reactions compete with each other: Cas12a destroys DNA (D), while the polymerase creates a new DNA molecule. The related kinetic equations are

(1) $$\frac{dC_a}{dt} = k_{1c}CD - k_{2c}C_a - k_cC_a$$

(2) $$\frac{dC}{dt} = -k_{1c}CD + k_{2c}C_a$$

(3) $$\frac{dP_a}{dt} = k_{1p}PD - k_{2p}P_a - k_pP_a$$

(4) $$\frac{dP}{dt} = - k_{1p}PD + k_{2p}P_a + k_pP_a$$

(5) $$\frac{dD}{dt} = - k_{1c}CD + k_{2c}C_a - k_{1p}PD + k_{2p}P_a + 2k_pP_a$$

We consider the quasi-equilibrium state wherein the formation and breakdown of the enzyme-substrate complex are in dynamic equilibrium; this state is reached shortly after the initial transient period when the reaction starts. For these steady states, we can approximate

(6) $$\begin{cases} \frac{dC_a}{dt} = k_{1c}CD - k_{2c}C_a - k_cC_a = 0 \\ \frac{dP_a}{dt} = k_{1p}PD - k_{2p}P_a - k_pP_a = 0 \end{cases}$$

which leads to

(7) $$\begin{cases} C_a = \frac{k_{1c}}{k_{2c} + k_c}CD = \frac{CD}{K_{Mc}} \\ P_a = \frac{k_{1p}}{k_{2p} + k_p}PD = \frac{PD}{K_{Mp}} \end{cases}$$

where K_{Mc} and K_{Mp} are the Michaelis-Menten (MM) constant for Cas12a *cis*-cleavage and the polymerase reaction, respectively. Using C_a and P_a in Eq. (7), we simplify Eq (5) as

(8) $$\frac{dD}{dt} = - \frac{k_c}{k_{Mc}}CD + \frac{k_p}{k_{Mp}}PD.$$

The first term on the right side describes the DNA loss due to Cas12a cleavage, while the second term is DNA increase from the polymerase reaction. The balance between these two terms decides the reaction fate – whether DNA will be amplified or entirely cleaved. This balance is particularly important at the initial phase of the CreDiT reaction when $C \approx C_0$ and $P \approx P_0$. Eq. (8) is approximated as

(9) $$\frac{dD}{dt} \approx - \underbrace{\frac{k_cC_0}{k_{Mc}}}_{a_c} D + \underbrace{\frac{k_pP_0}{k_{Mp}}}_{a_p} D$$

where a_c and a_p are the reaction rates of *cis*-cleavage and polymerization, respectively.

For the Cas12a *cis*-cleavage, the reported kinetic values are $k_{1c} = 1.3 \times 10^7$ M^{-1} s^{-1} (Ref. 15), $k_{2c} = 1.7 \times 10^{-3}$ s^{-1} (Ref. 15), and $k_c = 5 \times 10^{-3}$ s^{-1} (Refs. 16, 17), which gives $K_{Mc} = 5.2 \times 10^{-4}$ μM. For the polymerase reaction, the RPA kit uses polymerase I, whose kinetic parameters are estimated to be $k_{1p} = 4 \times 10^7$ M^{-1} s^{-1}, $k_{2p} = 0.2$ s^{-1}, and $k_p = 50$ s^{-1} (Ref. 18). These values give $K_{Mp} = 1.3$ μM. With $C_0 = 0.64$ μM and $P_0 = 1.3$ μM in our assay, the reaction rates are

	**Enzyme concentration (μM)**	**Turnover rate (s^{-1})**	**MM constant (μM)**	**Reaction rate (s^{-1})**
CAS12a *cis*	$C_0 = 0.64$	$k_c = 0.005$	$K_{Mc} = 5.2 \times 10^{-4}$	$a_c = 6.2$
Polymerase	$P_0 = 1.3$	$k_p = 50$	$K_{Mp} = 1.26$	$a_p = 52$

The DNA production is about 9-fold higher than the degradation by the Cas12a *cis*-cleavage activity. Based on the first-order kinetics (Eq. 9), the DNA amount will **exponentially increase over time**.

2. ***Trans*-cleavage of primers**

Activated Cas12a can interfere with DNA replication by indiscriminately cutting (*trans*-cleavage) RPA primers (single-stranded DNAs). However, kinetic considerations indicate that the polymerase reaction would dominate more than the Cas12a *trans*-cleavage reaction in CreDiT.

Following a similar procedure as in the *cis*-cleavage, the rate constant (a_t) of the *trans*-cleavage can be estimated as

$$(10)\quad a_t = \frac{k_t C_0}{k_{Mt}}$$

where k_t and K_{Mt} are the catalytic turnover rate and the MM constant for Cas12a *trans*-cleavage. We experimentally measured these values.

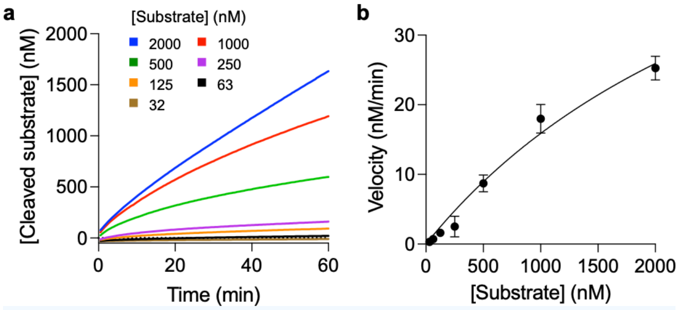

Supplementary Figure 22. Kinetics of Cas12a *trans*-cleavage. (a) Temporal increase of cleaved substrate. Fluorophore-quencher probes (used in CreDiT) were used as a substrate. The concentration of the activated Cas12a was fixed (1 nM), while the substrate concentration was varied. **(b)** The velocity of the enzyme reaction was obtained from (a) and fitted to an MM equation. The fitting results yielded k_t and K_{Mt}. Data are from technical triplicate and displayed as mean ± s.d.

Activated Cas12a (1 nM) was mixed with a substrate (fluorophore-quencher probe). The resulting fluorescent signals were monitored and converted into cleaved substrate concentrations (**Supplementary Fig. 22a**). From these curves, the velocity (V) of the enzymatic reaction was obtained and fitted to an MM equation (**Supplementary Fig. 22b**), which yielded $k_t = 1.2\ s^{-1}$ and $K_{Mt} = 3.4\ \mu M$.

The rate constants are now compared as

	**Enzyme concentration (μM)**	**Turnover rate (s^{-1})**	**MM constant (μM)**	**Reaction rate (s^{-1})**
CAS12a *trans*	$C_0 = 0.64$	$k_t = 1.2$	$K_{Mt} = 3.4$	$a_t = 0.2$
Polymerase	$P_0 = 1.3$	$k_p = 50$	$K_{Mp} = 1.3$	$a_p = 52$

Given this significant (>200-fold) difference in reaction rates, the polymerase is expected to outpace the Cas12a *trans*-cleavage activity, ensuring sufficient DNA amplification during the CreDiT assay.

SUPPLEMENTARY REFERENCES

1. Dai, Y. et al. Exploring the Trans-Cleavage Activity of CRISPR-Cas12a (cpf1) for the Development of a Universal Electrochemical Biosensor. *Angew Chem Int Ed Engl* **58**, 17399-17405 (2019).
2. Zhang, D. et al. CRISPR/Cas12a-Mediated Interfacial Cleaving of Hairpin DNA Reporter for Electrochemical Nucleic Acid Sensing. *ACS Sens* **5**, 557-562 (2020).
3. Liu, P. F. et al. Cas12a-based electrochemiluminescence biosensor for target amplification-free DNA detection. *Biosens Bioelectron* **176**, 112954 (2021).
4. Tsou, J. H., Leng, Q. & Jiang, F. A CRISPR Test for Detection of Circulating Nucleic Acids. *Transl Oncol* **12**, 1566-1573 (2019).
5. Omar Mukama, T. Y., Zhixu He, Zhiyuan Li, Jean de Dieu Habimana, Muzammal Hussain, Wei Li, Zhijian Yi, Qiongxin Liang, Lingwen Zeng. A high-fidelity CRISPR/Cas12a-based lateral flow biosensor for the detection of HPV16 and HPV18. *Sens Actuators B Chem* **316**, 128119 (2020).
6. Tang, Y. et al. CLIPON: A CRISPR-Enabled Strategy that Turns Commercial Pregnancy Test Strips into General Point-of-Need Test Devices. *Angew Chem Int Ed Engl.* **61**, e202115907 (2022).
7. Zhou, H. et al. Coupling CRISPR/Cas12a and Recombinase Polymerase Amplification on a Stand-Alone Microfluidics Platform for Fast and Parallel Nucleic Acid Detection. *Anal Chem* **95**, 3379-3389 (2023).
8. Yin, K. et al. Dynamic Aqueous Multiphase Reaction System for One-Pot CRISPR-Cas12a-Based Ultrasensitive and Quantitative Molecular Diagnosis. *Anal Chem* **92**, 8561-8568 (2020).
9. Teng, F. et al. CDetection: CRISPR-Cas12b-based DNA detection with sub-attomolar sensitivity and single-base specificity. *Genome Biol* **20**, 132 (2019).
10. Chen, J. S. et al. CRISPR-Cas12a target binding unleashes indiscriminate single-stranded DNase activity. *Science.* **360**, 436-439 (2018).
11. Xue, Y. et al. PddCas: A Polydisperse Droplet Digital CRISPR/Cas-Based Assay for the Rapid and Ultrasensitive Amplification-Free Detection of Viral DNA/RNA. *Anal Chem* **95**, 966-975 (2023).
12. Zhao, Y. et al. Integrating CRISPR-Cas12a into a Microfluidic Dual-Droplet Device Enables Simultaneous Detection of HPV16 and HPV18. *Anal Chem* **95**, 3476-3485 (2023).
13. Xu, Z. et al. Microfluidic space coding for multiplexed nucleic acid detection via CRISPR-Cas12a and recombinase polymerase amplification. *Nat Commun* **13**, 6480 (2022).
14. Cai, Y. et al. A dual-chamber "one-pot" CRISPR/Cas12a-based portable and self-testing system for rapid HPV diagnostics. *Sens Actuators, B* **405**, 135295 (2024).
15. Singh, D. et al. Real-time observation of DNA target interrogation and product release by the RNA-guided endonuclease CRISPR Cpf1 (Cas12a). *Proc Natl Acad Sci U S A* **115**, 5444-5449 (2018).
16. van Aelst, K., Martínez-Santiago, C. J., Cross, S. J. & Szczelkun, M. D. The Effect of DNA Topology on Observed Rates of R-Loop Formation and DNA Strand Cleavage by CRISPR Cas12a. *Genes (Basel)* **10**, 169 (2019).
17. Stella, S. et al. Conformational Activation Promotes CRISPR-Cas12a Catalysis and Resetting of the Endonuclease Activity. *Cell* **175**, 1856-1871.e21 (2018).
18. Kuznetsova, A. A., Fedorova, O. S. & Kuznetsov, N. A. Structural and Molecular Kinetic Features of Activities of DNA Polymerases. *Int J Mol Sci* **23**, 6373 (2022).
