# Supplementary material for: Empowering the on-site detection of nucleic acids by integrating CRISPR and digital signal processing
Source: Nat Commun. 2024 Jul 25;15:6271. doi: 10.1038/s41467-024-50588-3 (PMC11272939; doi:10.1038/s41467-024-50588-3)
Supplement: Supplementary file 3 — Description Of Additional Supplementary File [file 41467_2024_50588_MOESM3_ESM.pdf]

Description of Additional supplementary file

Supplementary Movie 1. Operation of the CreDiT system.

Supplementary Data 1. Clinical qPCR and CreDiT results for cervical cancer screening.
