# Supplementary material for: Empowering the on-site detection of nucleic acids by integrating CRISPR and digital signal processing
Source: Nat Commun. 2024 Jul 25;15:6271. doi: 10.1038/s41467-024-50588-3 (PMC11272939; doi:10.1038/s41467-024-50588-3)
Supplement: Supplementary file 6 — Reporting Summary [file 41467_2024_50588_MOESM6_ESM.pdf]

nature portfolio

Corresponding author(s): Cesar M. Castro, Hakho Lee

Last updated by author(s): Jul 12, 2024

Reporting Summary

Nature Portfolio wishes to improve the reproducibility of the work that we publish. This form provides structure for consistency and transparency in reporting. For further information on Nature Portfolio policies, see our Editorial Policies and the Editorial Policy Checklist.

Statistics

For all statistical analyses, confirm that the following items are present in the figure legend, table legend, main text, or Methods section.

n/a	Confirmed	
☐	☒	The exact sample size (*n*) for each experimental group/condition, given as a discrete number and unit of measurement
☐	☒	A statement on whether measurements were taken from distinct samples or whether the same sample was measured repeatedly
☐	☒	The statistical test(s) used AND whether they are one- or two-sided<br>*Only common tests should be described solely by name; describe more complex techniques in the Methods section.*
☒	☐	A description of all covariates tested
☒	☐	A description of any assumptions or corrections, such as tests of normality and adjustment for multiple comparisons
☐	☒	A full description of the statistical parameters including central tendency (e.g. means) or other basic estimates (e.g. regression coefficient) AND variation (e.g. standard deviation) or associated estimates of uncertainty (e.g. confidence intervals)
☐	☒	For null hypothesis testing, the test statistic (e.g. *F*, *t*, *r*) with confidence intervals, effect sizes, degrees of freedom and *P* value noted<br>*Give P values as exact values whenever suitable.*
☒	☐	For Bayesian analysis, information on the choice of priors and Markov chain Monte Carlo settings
☒	☐	For hierarchical and complex designs, identification of the appropriate level for tests and full reporting of outcomes
☐	☒	Estimates of effect sizes (e.g. Cohen's *d*, Pearson's *r*), indicating how they were calculated

Our web collection on statistics for biologists contains articles on many of the points above.

Software and code

Policy information about availability of computer code

Data collection	No software was used.
Data analysis	Programming language: R 4.2.2 R Core Team http://www.r-project.ofg/ and C++.<br>- The source code is available at https://github.com/deganii/fwht-arm<br>Quantification of images: Fiji imageJ and SciJava Projects http://imageJ.net/software/fiji/<br>Statistical analysis and graphing of data: Prism v9.5 GraphPad http://www.graphpad.com/scientificsoftware/prism/<br>Simulation of heating profile: COMSOL Multiphysics 5.6 https://www.comsol.com

For manuscripts utilizing custom algorithms or software that are central to the research but not yet described in published literature, software must be made available to editors and reviewers. We strongly encourage code deposition in a community repository (e.g. GitHub). See the Nature Portfolio guidelines for submitting code & software for further information.

Data

Policy information about availability of data

All manuscripts must include a data availability statement. This statement should provide the following information, where applicable:

- Accession codes, unique identifiers, or web links for publicly available datasets
- A description of any restrictions on data availability
- For clinical datasets or third party data, please ensure that the statement adheres to our policy

Source data are provided as a Source Data file.

Research involving human participants, their data, or biological material

Policy information about studies with human participants or human data. See also policy information about sex, gender (identity/presentation), and sexual orientation and race, ethnicity and racism.

Reporting on sex and gender	Cervical brush samples were collected from female participants (n = 121). The sample sizes were 69 for HPV positives and 52 for HPV negatives. Anal swab samples were collected from female (n =13) and male (n = 35) participants. The gender of participants was determined based on self-reported information provided on the study consent form.
Reporting on race, ethnicity, or other socially relevant groupings	Race/ethnicity was not considered as an explanatory variable in data analyses. The race/ethnicity of participants was determined based on self-reported information provided on the study consent form.
Population characteristics	For cervical cancer detection, we obtained cervical brushing specimens collected during routine gynecologic evaluation. For anal cancer detection, we obtained anal brushing specimens collected during anal exams. The summaries of participants characteristics are shown in Table 1 (cervical) and Supplementary Table 5 (anal).
Recruitment	We enrolled the first subjects who met the eligibility criteria. Children defined as aged less than 18 were excluded from the study. Otherwise, there were no specific exclusion criteria. We have obtained informed consent from all participants, and this is noted in the manuscript.
Ethics oversight	The research protocol received approval from the Partners Healthcare Institutional Review Board (IRB protocol 2022P002938).

Note that full information on the approval of the study protocol must also be provided in the manuscript.

Field-specific reporting

Please select the one below that is the best fit for your research. If you are not sure, read the appropriate sections before making your selection.

☒ Life sciences ☐ Behavioural & social sciences ☐ Ecological, evolutionary & environmental sciences

For a reference copy of the document with all sections, see nature.com/documents/nr-reporting-summary-flat.pdf

Life sciences study design

All studies must disclose on these points even when the disclosure is negative.

Sample size	The sample size was not predetermined, but our sample number is powered to determine whether the CreDiT test offers high diagnostic accuracy. We used the area under the ROC curve (AUC) as an index of accuracy. Our null hypothesis was that the CreDiT test has an AUC of 0.7 (a fair test), whereas the alternative hypothesis was that the CreDiT test performs significantly better (AUC = 0.9, a highly accurate test). The sample size we used (≥16 HPV-target positives and ≥16 HPV-target negatives) achieves at least 89% power to detect this AUC difference of 0.2 at a significance level of 5% (one-sided test).
Data exclusions	No data were excluded from the analyses.
Replication	All clinical samples were measured at least triplicate (technical replica) for each DNA (for HPV diagnosis and subtyping) and mRNA marker (for expression level of oncoprotein), and the mean values were used for analyses. All attempts at replication were successful.
Randomization	Not relevant. On a rolling basis, we recruited participants who came first, met the eligible criteria, and gave informed consent.
Blinding	We analyzed the clinical samples using the CreDiT assay while blinded to the clinical diagnostic results (gold standard).

Reporting for specific materials, systems and methods

We require information from authors about some types of materials, experimental systems and methods used in many studies. Here, indicate whether each material, system or method listed is relevant to your study. If you are not sure if a list item applies to your research, read the appropriate section before selecting a response.

Materials & experimental systems

n/a	Involved in the study
☒	☐ Antibodies
☐	☒ Eukaryotic cell lines
☒	☐ Palaeontology and archaeology
☒	☐ Animals and other organisms
☒	☐ Clinical data
☒	☐ Dual use research of concern
☒	☐ Plants

Methods

n/a	Involved in the study
☒	☐ ChIP-seq
☒	☐ Flow cytometry
☒	☐ MRI-based neuroimaging

Eukaryotic cell lines

Policy information about cell lines and Sex and Gender in Research

Cell line source(s)	Ca SKi (CRL-1550), SiHA (HTB-35), HeLa (CCL2), and C-33 A (HTB-31) cells were purchased from the American Type Culture Collection (ATCC) within the last two years. All cervical cancer cell lines were originally derived from female donors.
Authentication	All cell lines had been authenticated by the ATCC. We used cells without any modification.
Mycoplasma contamination	We tested cell lines for mycoplasma contamination (MycoAlert Mycoplasma Detection Kit, Lonza, LT07-418). All cell lines used in this work were free of mycoplasma contamination.
Commonly misidentified lines (See ICLAC register)	None

Plants

Seed stocks	*Report on the source of all seed stocks or other plant material used. If applicable, state the seed stock centre and catalogue number. If plant specimens were collected from the field, describe the collection location, date and sampling procedures.*
Novel plant genotypes	*Describe the methods by which all novel plant genotypes were produced. This includes those generated by transgenic approaches, gene editing, chemical/radiation-based mutagenesis and hybridization. For transgenic lines, describe the transformation method, the number of independent lines analyzed and the generation upon which experiments were performed. For gene-edited lines, describe the editor used, the endogenous sequence targeted for editing, the targeting guide RNA sequence (if applicable) and how the editor was applied.*
Authentication	*Describe any authentication procedures for each seed stock used or novel genotype generated. Describe any experiments used to assess the effect of a mutation and, where applicable, how potential secondary effects (e.g. second site T-DNA insertions, mosiacism, off-target gene editing) were examined.*
